# Sperm-Associated Antigen 6 (SPAG6) Deficiency and Defects in Ciliogenesis and Cilia Function: Polarity, Density, and Beat

**DOI:** 10.1371/journal.pone.0107271

**Published:** 2014-10-21

**Authors:** Maria E. Teves, Patrick R. Sears, Wei Li, Zhengang Zhang, Waixing Tang, Lauren van Reesema, Richard M. Costanzo, C. William Davis, Michael R. Knowles, Jerome F. Strauss, Zhibing Zhang

**Affiliations:** 1 Department of Obstetrics and Gynecology, Virginia Commonwealth University, Richmond, Virginia, United States of America; 2 Department of Biochemistry and Molecular Biology, Virginia Commonwealth University, Richmond, Virginia, United States of America; 3 Cystic Fibrosis Center, University of North Carolina, Chapel Hill, North Carolina, United States of America; 4 Department of Infectious Diseases, Tongji Medical College, Huazhong University of Science and Technology, Wuhan, Hubei, China; 5 Department of Otorhinolaryngology, University of Pennsylvania, Philadelphia, Pennsylvania, United States of America; 6 Department of Physiology and Biophysics, Virginia Commonwealth University, Richmond, Virginia, United States of America; 7 Department of Cell & Molecular Physiology of Medicine, University of North Carolina, Chapel Hill, North Carolina, United States of America; University of Illinois at Chicago, United States of America

## Abstract

SPAG6, an axoneme central apparatus protein, is essential for function of ependymal cell cilia and sperm flagella. A significant number of *Spag6*-deficient mice die with hydrocephalus, and surviving males are sterile because of sperm motility defects. In further exploring the ciliary dysfunction in *Spag6*-null mice, we discovered that cilia beat frequency was significantly reduced in tracheal epithelial cells, and that the beat was not synchronized. There was also a significant reduction in cilia density in both brain ependymal and trachea epithelial cells, and cilia arrays were disorganized. The orientation of basal feet, which determines the direction of axoneme orientation, was apparently random in *Spag6*-deficient mice, and there were reduced numbers of basal feet, consistent with reduced cilia density. The polarized epithelial cell morphology and distribution of intracellular mucin, α-tubulin, and the planar cell polarity protein, Vangl2, were lost in *Spag6*-deficient tracheal epithelial cells. Polarized epithelial cell morphology and polarized distribution of α-tubulin in tracheal epithelial cells was observed in one-week old wild-type mice, but not in the *Spag6*-deficient mice of the same age. Thus, the cilia and polarity defects appear prior to 7 days post-partum. These findings suggest that SPAG6 not only regulates cilia/flagellar motility, but that in its absence, ciliogenesis, axoneme orientation, and tracheal epithelial cell polarity are altered.

## Introduction

Mammalian SPAG6 is the orthologue of PF16, a component of the central apparatus of the “9+2” axoneme of the green algae model organism, *Chlamydomonas reinhardtii* [1]. In *Chlamydomonas*, PF16 protein is present along the length of the flagella, and immunogold labeling localizes the PF16 protein to a single microtubule of the central pair. Mutations in the *Chlamydomonas* PF16 gene cause flagellar paralysis, and PF16 is believed to be involved in C1 central microtubule stability and flagellar motility [2]. In addition to *Chlamydomonas reinhardtii*, SPAG6/PF16 has been shown to regulate flagellar motility in other models, including trypanosomes, Plasmodium, and Giardia [3], [4], [5].

Gene targeting has been used to create mice lacking SPAG6 [6]. Approximately 50% of *Spag6*-deficient animals died from hydrocephalus before adulthood, and males surviving to maturity were infertile. Even though an abnormal axoneme ultrastructure was discovered in the *Spag6*-deficient sperm [6], cilia of brain ependymal cells and trachea epithelial cells from the mutant mice contained “9+2” axonemes that appeared to be grossly intact [7]. However, brain ependymal cells of *Spag6*-deficient mice are functionally defective since hydrocephalus develops.

In further characterizing the cilia abnormalities of *Spag6*-deficient mice, we discovered that ciliary beat frequency was significantly reduced. The mutant mice also had fewer trachea and ependymal cilia, and these cilia were arrayed in a random fashion on the cell surface. The central pair orientation differed significantly between cilia of the *Spag6*-deficient mice, reflecting the random orientation of basal feet. The polarized epithelial cell morphology and distribution of α-tubulin and planar cell polarity protein, Vangl2, were lost in *Spag6*-deficient tracheal epithelial cells. These findings suggest that mouse SPAG6 has multiple functions: it regulates cilia/flagellar motility through the central pair apparatus; but also plays a role in ciliogenesis, axoneme orientation, and cell polarity.

## Materials and Methods

### *Spag6* and *Spag16L* mutant mice

*Spag6* and *Spag16L* mutant mice were generated previously in our laboratory [6], [8]. All animal work was approved by Virginia Commonwealth University's Institutional Animal Care & Use Committee (protocol #AM10297 and AD10000167) in accordance with Federal and local regulations regarding the use of non-primate vertebrates in scientific research.

### High-speed video analysis of ciliary beat frequency

Ciliary beat frequency was assessed with the Sisson–Ammons video analysis (SAVA) system (Ammons Engineering, Mt. Morris, MI) [9]. Tracheas from wild type and *Spag6*-deficient mice (3 weeks old) were removed and video movies were taken within five minutes with an Nikon Eclipse TE-2000 inverted microscope (×40 phase-contrast objective) equipped with an ES-310 Turbo monochrome high-speed video camera (Redlake, San Diego, CA) set at 125 frames per second. The ciliary beat pattern was evaluated on slow-motion playbacks.

### Transmission electron microscopy

For transmission electron microscopy (TEM), the samples (from 3 week old mice) were cut into small sections (2×2 mm) and fixed in 2.5%glutaraldehyde (PH = 7. 4) for 6–8 hours at 4°C. They were washed and post fixed in 2% OsO4 for 1 hour, at 4°C. The tissue was dehydrated through ascending series of ethanol concentrations and embedded in araldite CY212. Semi thin sections (1 µm) were cut and stained with toluidine blue. Ultra-thin sections (60–70 nm) were cut and stained with uranyl acetate and alkaline lead citrate.

### Scanning electron microscopy

Specimens (from 3 week old mice) were fixed with 1.5% glutaraldehyde and 1.5% paraformaldehyde in 0.1 M sodium phosphate buffer, pH 7.3 for 3 hours at room temperature and postfixed for two hours in 2% osmium tetroxide in 0.1 sodium phosphate buffer. After dehydration in graded ethanol, samples for scanning electron microscopy (SEM) were dried in a critical-point dryer (Polaron, Watford, UK), mounted on stubs, and coated with gold-palladium in a cool sputter coater (Fisons Instruments Uckfield, UK). The specimens were examined using a scanning electron microscope DSM 960 (Zeiss Oberkochen, Germany).

### Histology

H&E and Periodic acid-Schiff (PAS) staining on mouse trachea (from 1 and 3 week old mice) were carried out using standard procedures. 5 µm sections were cut for experiments.

### Immunofluorescence staining of brain and trachea

Brain and trachea from wild-type and *Spag6*-mutant mice (3 week old) were fixed with 4% paraformaldehyde in 0.1 M PBS (pH 7.4), and 5 µm paraffin sections were made. For the immunofluorescence, the method described by Tsuneoka was used [10]. The sections were incubated with an anti-Vangl2 or anti-acetylated tubulin primary antibody at 4°C for overnight. Slides were washed with PBS and incubated for 1 hour at room temperature with Alexa 488-conjugated anti-mouse IgG secondary antibody (1∶500; Jackson ImmunoResearch Laboratories) or Cy3-conjugated anti-rabbit IgG secondary antibody (1∶5000; Jackson ImmunoResearch Laboratories). Following secondary antibody incubation, the slides were washed again three times in PBS, mounted using VectaMount with 4′, 6-diamidino-2-phenylindole (DAPI) (Vector Laboratories, Burlingame, CA), and sealed with a cover slip. Images were captured by confocal laser-scanning microscopy (Leica TCS-SP2 AOBS).

### Video-microscopy

Tracheas were collected from three week old wild-type and *Spag6* knockout mice. Trachea sections were placed luminal side down on a coverslip containing some blood drops in 37°C PBS. Cilia movement and blood cells flows were observed with differential interference contrast microscopy using an inverted microscope (Nikon) equipped with a 100 X oil immersion objective. Images were recorded at 30 frames per second with SANYO color CCD, Hi-resolution camera (VCC-3972, Sanyo Electric Co, Japan) and Pinnacle Studio HD (Ver. 14.0, Pinnacle Systems, Inc., Mountain View, CA, USA) software. Several randomly selected areas were imaged for each sample. Quantification of blood cells directionality was performed with ImageJ software and plugin MTrackJ (NIH). 200 blood cells were tracked for each sample. Directionality was defined as the net displacement achieved divided by the total distance traveled. A directionality of 1 indicated the blood cell moved in a straight line, while a directionality of 0 represents a random movement approach. Data represent mean ± SEM of three mice for each genotype.

### Western blot analysis

Equal amounts of protein (50 µg/lane) were heated to 95°C for 10 minutes in sample buffer, loaded onto 10% sodium dodecyl sulfate-polyacrylamide gels, electrophoretically separated, and transferred to polyvinylidene difluoride membranes (Millipore, Billerica, MA). Membranes were blocked (Tris-buffered saline solution containing 5% nonfat dry milk and 0.05% Tween 20 (TBST)) and then incubated overnight with indicated antibodies at 4°C. After washing in TBST, the blots were incubated with second antibodies for 1 hour at room temperature. After washing, the proteins were detected with Super Signal chemiluminescent substrate (Pierce, Rockford, IL).

### Quantitative analysis of basal foot orientation

A reference line was drawn for each image. For each basal foot, a vector connecting the center of the basal body and the protrusion of the basal foot was drawn. The angle between this vector and the reference line was measured manually using ImageJ software (NIH). Five images were analyzed from each mouse, and three wild-type and three mutant mice were used for the analysis. The mean angle was calculated for each cell using Oriana 4.0 software (Kovach Computing Services). The mean angle was defined as mean ciliary direction (shown as 0° in each circular plot graph). Deviation from the mean angle was calculated for all of the basal feet analyzed. Deviation angles of the basal feet were pooled and plotted on a circular graph using Oriana 4.0 software.

### Statistical methods

Significant difference of axoneme number and basal feet number between wild-type and *Spag6*-deficient mice was calculated using student *t* test. Significant difference of CBF among wild-type, *Spag6*, and *Spag16L*-deficient mice was calculated using ANOVA. * = significant at 0.05. Statistical analysis of tracheal epithelial cell basal body rootlet orientation was carried out with Oriana 4.0 (Kovach Computing Services) circular statistics software.

## Results and Discussion

A recent study reported that the mouse has two copies of the *Spag6* gene, the one previously studied on chromosome 16, which is proposed to have evolved from the parental isoform, *Spag6-BC061194*, which is located on chromosome 2 [11]. Even though the amino acid sequences of the two SPAG6 proteins are 97% identical, the nucleotide sequences of the two *Spag6* genes are significantly different. We confirmed that the *Spag6*-deficient mouse we created and studied retains the *Spag6*-BC061194 gene, so that the phenotypes we have described represent the solely the impact of the loss of the “evolved” *Spag6* gene (data not shown).

**Figure 1:**
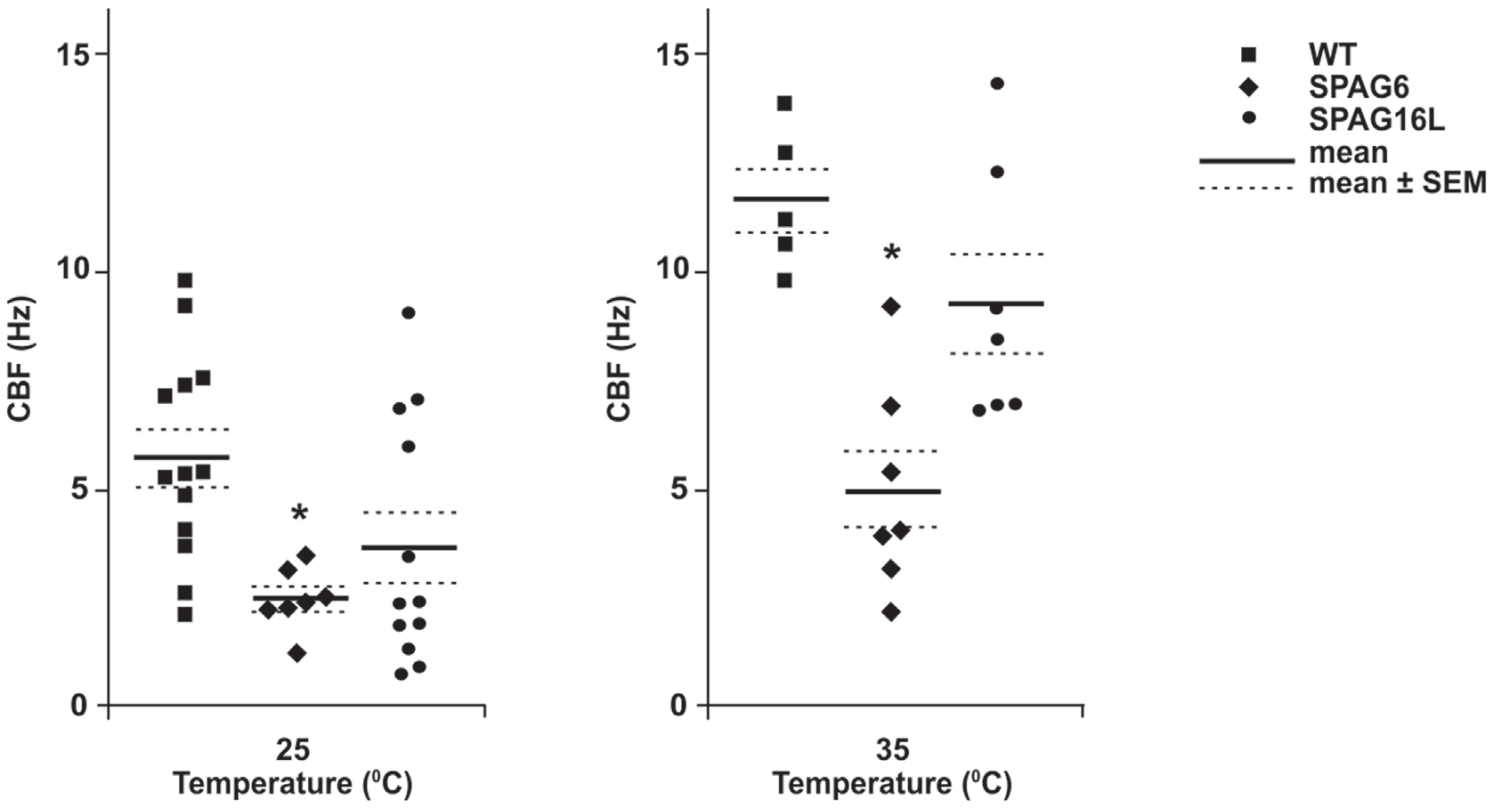
Trachea ciliary beat frequency (CBF) is dramatically decreased in *Spag6*-mutant mice. Graph showing ciliary beat frequency for wild-type, *Spag6*, and *Spag16L* mutant mice. The mean CBF in *Spag6*-deficient mice was significantly lower than that in the wild type and *Spag16L* mice at both 25°C (n = 13, 7 and 12 for wild-type, *Spag6* mutant, and *Spag16L* mutant mice respectively) and 35°C (n = 5, 7 and 7 for wild-type, *Spag6* mutant, and *Spag16L* mutant mice respectively). *p<0.05. ANOVA was conducted to determine significant difference.

Ciliary beat frequency (CBF) of tracheal cilia was measured in *Spag6*-deficient and littermate wild-type mice. Compared to the wild-type mice, baseline CBF was significantly reduced in the *Spag6*-deficient mice at both room temperature (25°C) and 35°C (Fig. 1). Of note, mutation of the *Spag16L* gene, which encodes a central apparatus protein, SPAG16L, that interacts with SPAG6, does not cause CBF abnormalities or uncoordinated cilia beat [12], despite the fact that *Spag16L* mutant mice are infertile due to a severe sperm motility defect [8]. Thus, SPAG6 has functional roles in tracheal cilia that are distinct from those of SPAG16L.

**Figure 2:**
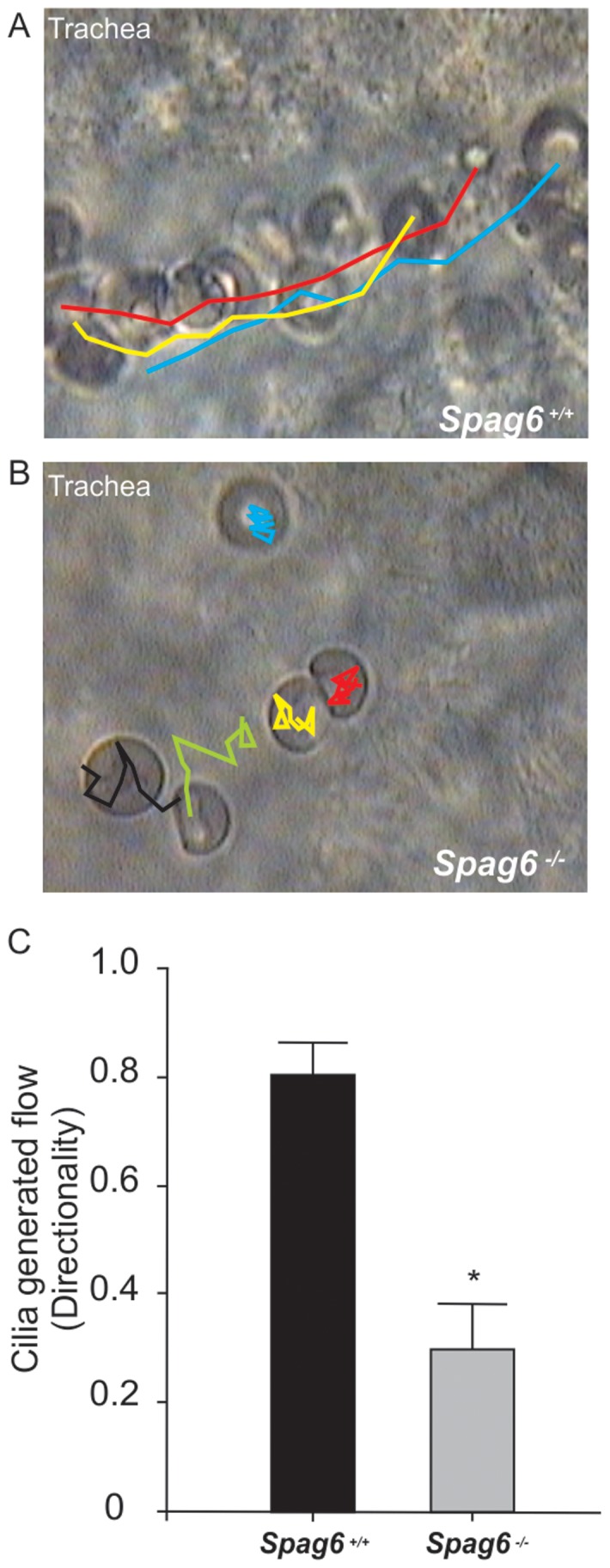
Cilia-generated flow is significantly reduced in *Spag6*-deficient tracheal epithelium. A) Longitudinal view of tracheal epithelia from wild-type mouse showing the tracking of movement of blood cells. B) Longitudinal view of tracheal epithelia from *Spag6* knockout mouse showing the tracking of movement of blood cells. C) Cilia generated flow was quantified by analyzing the directionality of movement of blood cells. * Significant differences (p<0.05) vs. wild-type. Data are presented as mean ± SEM. The colors indicates movement track of individual blood cells.

Tracheal ciliary beating was also observed by video microscopy. Consistent with the CBF results, cilia from wild-type mice beat at a faster rate, and the beat was coordinated, with all the cilia beating in the same direction at a specific time point (Video S1). The metachronal beating resulted in a directional flow as shown by the movement of particles/blood cells (Video S2 and Fig. 2A). However, cilia from *Spag6*-deficient mice beat at a much slower rate, and the beating was largely uncoordinated. At specific time points, some cilia beat in one direction, but others in an opposite direction (Video S3). Significantly reduced directed flow was observed as the blood cells collected at the beginning of the tracheal tubes (Video S4, Fig. 2B and Fig. 2C).

**Figure 3:**
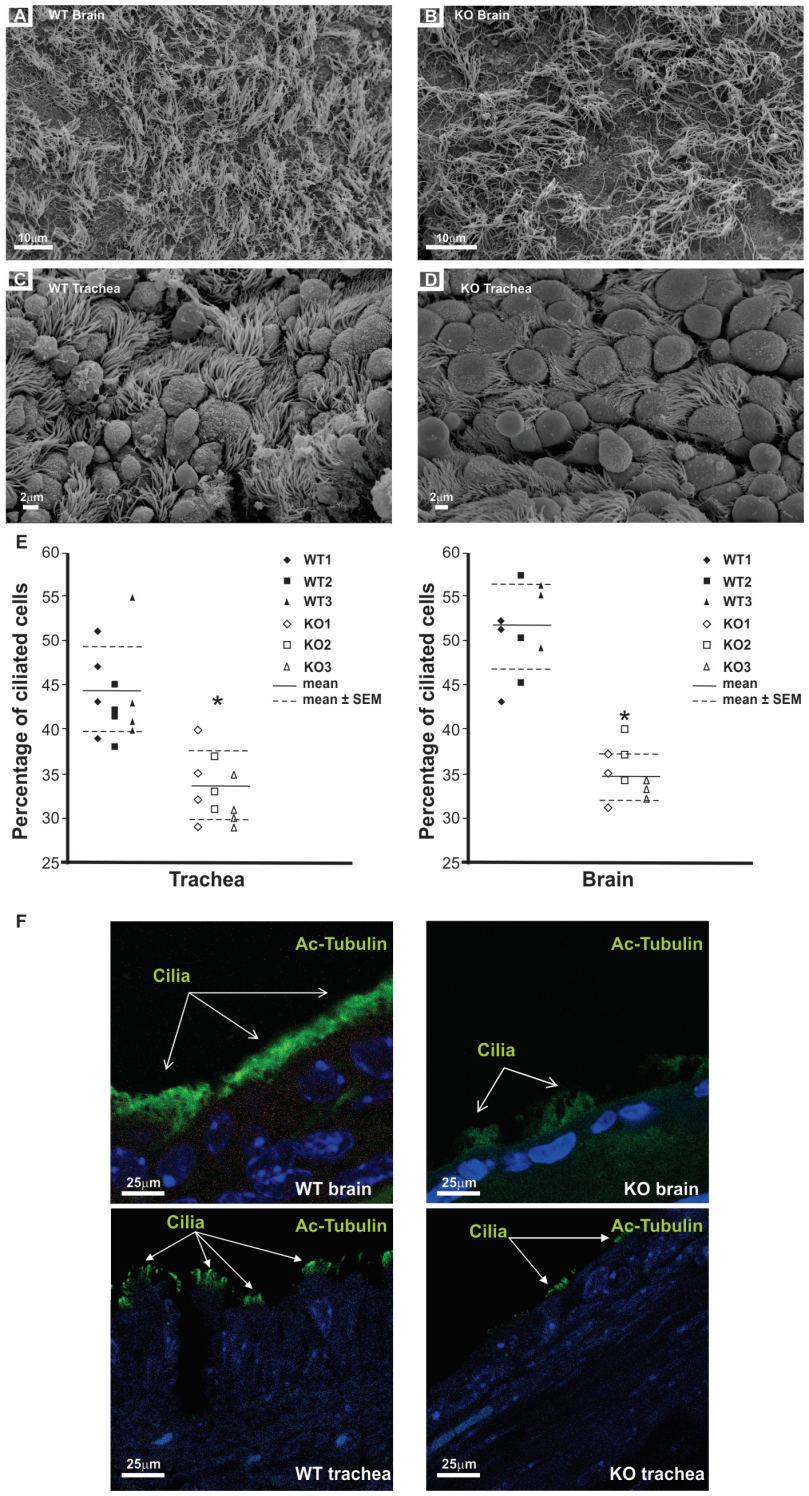
Analyses of cilia in the trachea epithelial cells and brain ependymal cells by scanning electronic microscopy and immunofluorescence staining. Representative images from SEM analyses from wild-type and *Spag6*-deficient mice. Cilia of the wild type mice were well-organized in both brain ependymal cells (A) and trachea epithelial cells (C). In contrast, there was a dramatic reduction in cilia density in both brain ependymal cells (B) and trachea epithelial cells (D) of the *Spag6*-deficient mice, and the cilia were disorganized. To calculate the percentage of ciliated cells, cells with cilia and total cells were counted from three (brain) or four (trachea) SEM images from each mouse, and ratio was calculated (E). Three wild-type and three *Spag6*-deficient mice were analyzed. * Significant differences vs. wild-type (p<0.05). Brain and trachea sections from wild type and *Spag6*-deficient mice were examined by immunofluorescence staining using an antibody targeting acetylated tubulin. In the wild type mice, the cilia-containing signal was continuously observed along the surface of the epithelial cells. However, in the *Spag6*-deficient mice, the signal was discontinuous (F).

Scanning electron microscopy (SEM) was carried out on three (3 week old) wild-type and three *Spag6*-deficient mice of the same age to examine cilia orientation. Tracheal and ependymal cilia in the wild-type animals are anchored to the cell surface in organized arrays (Fig. 3A, 3C). In contrast, cilia arrays of *Spag6*-deficient mice were disorganized (Fig. 3B, 3D, Figure S1C and Figure S1D). In addition, there was a dramatic reduction in cilia density in both brains and tracheas of the *Spag6*-deficient mice, and the percentage of ciliated cells was significantly lower in the mutant mice (Fig. 3E). Cilia in these tissues were further examined by immunofluorescence staining using an antibody to acetylated tubulin. In wild-type mice, the cilia signal was continuous along the surface of the epithelial cells, and extended away from the cell surface into the lumen (Fig. 3F, left panel). However, in the *Spag6*-deficient mice, the signal was discontinuous, and the signal extended to a lesser extent from cell surface than in wild-type tissues (Fig. 3F, right panel).

**Figure 4:**
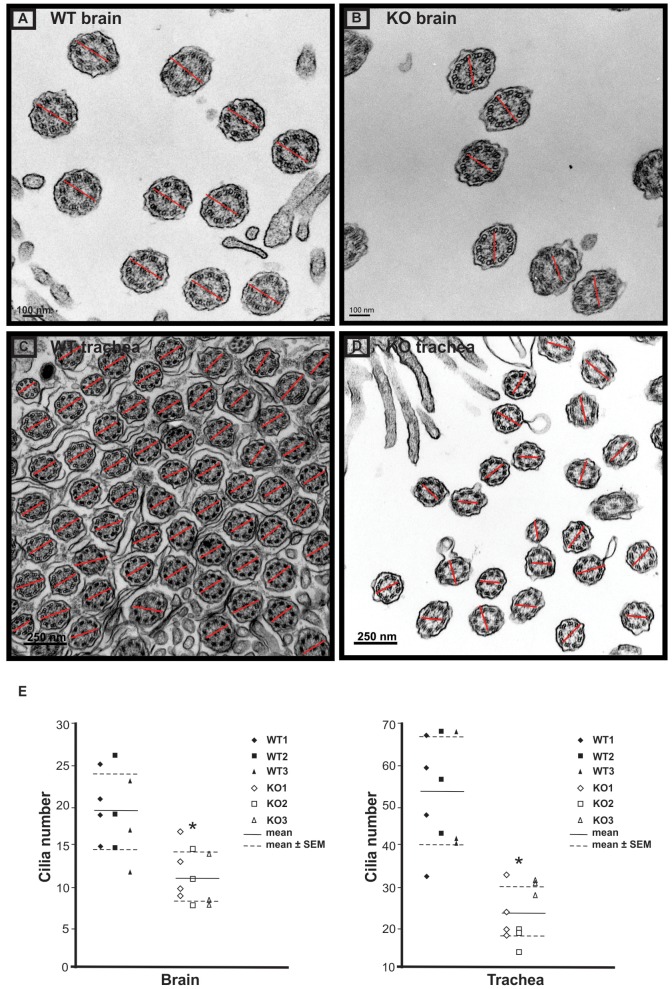
Examination of rotational polarity of ciliary axoneme of brain ependymal cells and trachea epithelial cells by transmission electronic miscroscopy. Axoneme cross-sectional images were taken with a transmission electron microscope. The rotational polarity of each axoneme was evaluated by the angle of the line connecting the central pair. Notice that the orientation of the lines in wild type mice is similar (A: brain; C: trachea). while the orientation of the lines in the *Spag6*-deficient mice varies among axonemes (B: brain; D: trachea). E. Average axoneme number counted from ten images randomly selected from each group. Three or four images were counted from each mouse, and three wild-type and three mutant mice were examined. Horizontal lines represent the means and SEMs. * p<0.05.

Transmission electron microscopy (TEM) was conducted to examine the orientation of the central pair microtubules in four wild-type and four *Spag6*-deficient mice. In wild-type animals, orientation of the two central microtubules of all the cilia in the ependymal cells (Fig. 4A) and tracheal epithelial cells (Fig. 4C) was consistent, as shown by the similar orientation of lines connecting the two microtubules in all the axonemes. In the *Spag6*-deficient mice, the axoneme structure appeared normal, but the orientation of the two central microtubules was random; lines connecting central microtubules pointed to one direction in some axonemes, while the lines pointed to a different direction in others (Fig. 4B, and Fig. 4D). To compare the cilia number in the brain ependymal and trachea epithelial cells of wild-type and *Spag6*-deficient mice, the axoneme number was counted from ten TEM images randomly selected from each group. The *Spag6*-deficient mice had significantly lower axoneme numbers than that in the wild-type mice (p<0.05, Fig. 4E).

**Figure 5:**
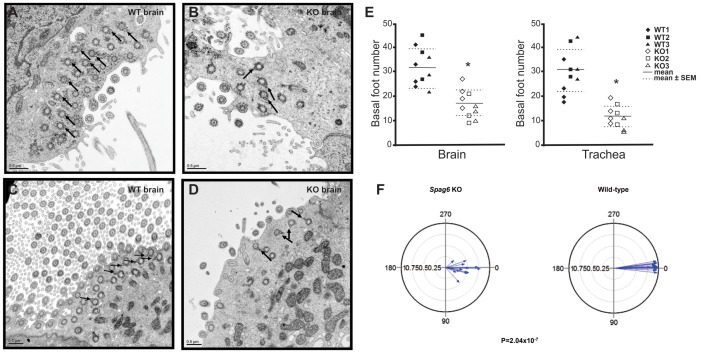
Basal feet polarity of brain ependymal cells and trachea epithelial cells was lost in the *Spag6*-deficient mice. Basal body images were taken with a transmission electron microscope. Notice that the basal feet point to the same orientation in the wild type animals (A: brain; C: trachea). However, they point to different orientation in the *Spag6*-deficient mice (B: brain; D: trachea). The number of basal body in the *Spag6*-deficient mice was significantly reduced in both brain and trachea. The arrows point to the basal feet. E. Average basal feet number counted from ten images randomly selected from each group. Horizontal lines represent means and SEMs. Three or four images were counted from each mouse, and three wild-type and three mutant mice were examined. * p<0.05. F. Circular plots of tracheal epithelial cell basal feet orientation in *Spag6*-deficient (left) and wild-type mice (right). For each mouse, basal foot orientation from five images was analyzed. For each image, the angel for one basal foot orientation was set as 0° (or 360°), angels of the rest basal feet were measured. Each plot represents the combined data from three mice as shown in Figure S2 (* p<0.001 between the two groups).

The ciliary beat orientation is determined by the orientation of the basal feet [13]. The basal feet were examined in the brain ependymal and trachea epithelial cells of wild-type and the *Spag6*-deficient mice by TEM. In the wild-type mice, the basal feet were present in both brain ependymal cells (Fig. 5A) and trachea epithelial cells (Fig. 5C), and they were organized in a similar orientation. However, in the *Spag6*-deficient mice, even though basal feet were morphologically intact, the orientation was random. Like the orientation of the central microtubules in the *Spag6*-deficient mice, some basal feet pointed to one direction, others pointed in different direction (Fig. 5B, and Fig. 5D). The basal feet number was also counted from the TEM images, and the number was significantly reduced in both brain ependymal (p<0.05) and trachea epithelial cells (p<0.05) of the *Spag6*-deficient mice (Fig. 5E). To determine if the organization of basal feet in *Spag6*-mutant mice is significantly different compared to wild-type animals, basal foot orientation of tracheal epithelial cells was analyzed in three *Spag6* mutant mice (Figure S2A) and three wild-type mice (Figure S2B). Statistical analysis demonstrated that there was significant difference in the *r*_cell_ metric between the mutant and wild-type mice (Fig. 5F), suggesting that intracellular planar polarity was lost in the mutant mice.

**Figure 6:**
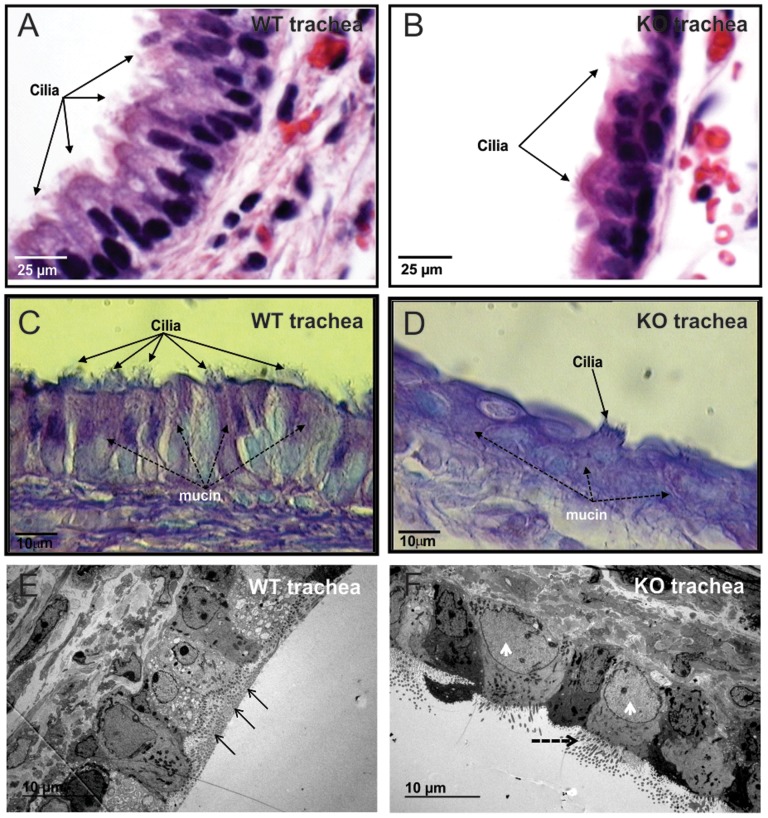
Examination of trachea epithelial cell polarity in three-week old mice. H&E stained tissue demonstrating that two or three rows of nuclei were seen in the pseudostratified columnar epithelium lining the trachea of the wild-type mice, and the nuclei were oval shape and the two poles were at basal/apical distribution (Fig. 6A). In contrast, in the *Spag6*-deficient mice, the cells lie flat along the basement membrane, most cells had round nuclei (Fig. 6B). PAS staining demonstrated that mucin was localized in apical region of epithelial cells (Dashed arrows in Fig. 6C). However, this pattern was never seen in the epithelial cells of *Spag6*-deficient mice, mucin was present through the whole cytoplasm (Fig. 6D). The above-mentioned differences between wild-type and *Spag6*-deficient mice were confirmed by TEM with low magnification. The wild-type epithelial cells show polarized pattern, and mucin was found along the surface in all the three mice analyzed (arrows in Fig. 6E), where the cilia axonemes were located. However, in the mutant mice, the epithelial cells lost this pattern, and the cells look larger than those in the wild-type mice (arrow heads), and no mucin was found in any of the three mice analyzed (Fig. 6F). Three wild-type and three mutant mice were analyzed and the results were similar.

To investigate tissue-level cell polarity, histological sections of tracheas from three (3 week old) wild type and three *Spag6*-deficient mice were examined by light microscopy. H&E staining revealed that in the wild-type mice, two or three rows of nuclei were present in the pseudostratified columnar epithelium lining of the trachea, and the nuclei were oval in shape and oriented in a basal/apical distribution (Fig. 6A). However, the *Spag6*-deficient epithelial cells did not form the pseudostratified columnar morphology. Only one row of nuclei was present, and the cells lay relatively flat along the basement membrane, with most cells having round nuclei (Fig. 6B). The *Spag6*-deficient tracheal epithelial cells not only lose the polarized morphology, the polarized distribution of mucin was also absent. In the wild-type mice, PAS staining demonstrated that mucin was localized in apical region of epithelial cells (Fig. 6C). However, this pattern was never seen in the epithelial cells of *Spag6*-deficient mice. In contrast, mucin was present throughout the cytoplasm (Fig. 6D). The polarized morphology of wild-type tracheal epithelial cells was also observed in low magnification TEM images, and mucin was observed on the trachea surface (Fig. 6E). However, the polarized pattern was lost in the mutant mice, and mucin was not detected on the surface of the tracheal epithelial cells (Fig. 6F).

The localization of the planar cell polarity protein, Vangl2, was examined in the tracheas of three wild-type and three *Spag6*-deficient mice by immunofluorescence staining. Even though there was no difference in total expression level of the protein in trachea/lung between wild-type and the *Spag6*-deficient mice by Western blot analysis (Figure S3), it appears that in wild-type trachea epithelial cells from three-week old mice, Vangl2 signal was more intense in the apical region (Figure S4A). This polarized distribution was not evident in the *Spag6*-deficient epithelial cells (Figure S4B).

Immunofluorescence staining was also conducted on tracheas from three week old wild-type and age-matched *Spag6*-deficient mice using an anti-α-tubulin antibody. In the trachea of wild-type mice, cilia were intensely stained. Inside the epithelial cells, a strong signal was visualized in the apical regions (Figure S4C). However, in the *Spag6*-deficient mice, the signal was evenly distributed throughout the whole cell body (Figure S4D). The microtubule distribution pattern is consistent with that of the planar cell polarity (PCP) proteins, suggesting that polarized microtubules might contribute to the localization of PCP proteins.

**Figure 7:**
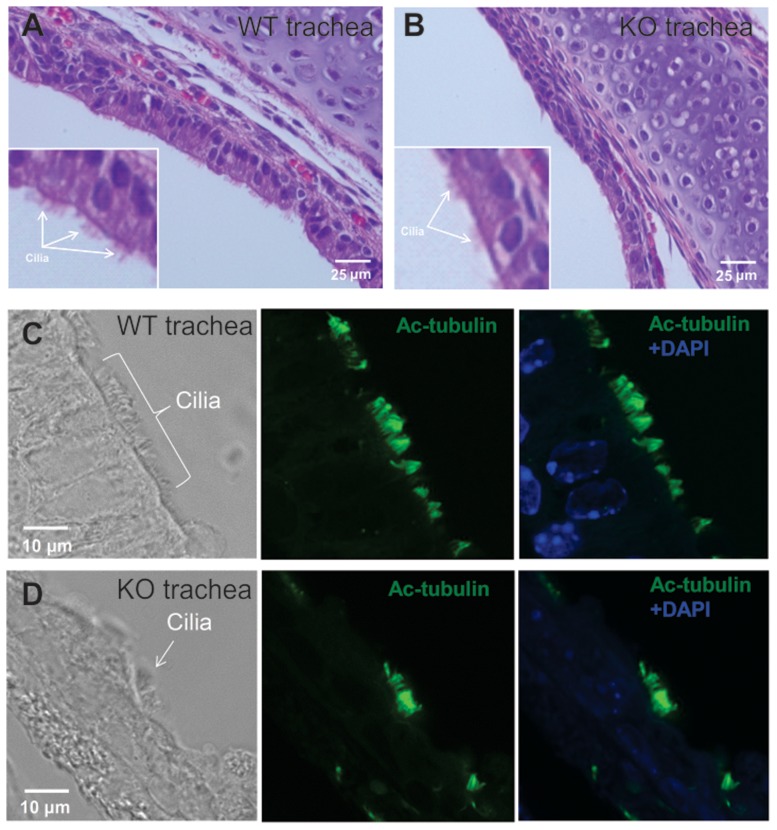
Examination of trachea epithelial cell polarity in one-week old mice. H&E stained tissue demonstrating the polarized pattern of epithelial cells in the trachea of wild type mice (Fig. 7A), but not in the *Spag6*-deficient mice (Fig. 7B). Acetylated tubulin signal is abundant along the tracheal epithelial cells in the wild-type mice (Fig. 7C), the signal is dramatically reduced in the *Spag6*-deficient mice (Fig. 7D). Three wild-type and three mutant mice were analyzed and similar results were observed.

One-week mice were analyzed, when the mutant mice did not show obvious abnormalities related to reduced ciliary motility, such as hydrocephalus. As in the three-week old wild-type mice, epithelial cells in the one-week old wild-type mice are polarized as shown by H&E staining (Fig. 7A), and immunofluorescence staining using an anti-acetylated tubulin antibody revealed a strong cilia signal (Fig. 7C). However, the epithelial cells of the *Spag6*-deficient mice did not show the polarized morphology. Compared to the wild-type mice, cilia number is dramatically reduced (Fig. 7B, 7D). The polarized distribution of α-tubulin is obvious in the wild-type mice (Figure S5A), but not in the *Spag6*-deficient mice (Figure S5B). These findings indicate that the cilia number and orientation defects are present earlier than 7 days post-partum.

It has been reported that a feedback loop generated by fluid flow contributes to cilia polarization [14], [15]. Indeed, our findings of disorganization and reduced number of cilia in ependymal and tracheal epithelial cells in *Spag6*-mutants, and alterations in basal body alignment are similar to those previously described in other mutant mice as a consequence of reduced ciliary motility. Studies involving Jhy^lacZ/lacZ^ mice showed disorganization and altered axonemal structure of the ependymal cilia. However, the hydrocephalus appeared to be unrelated to abnormal brain development or patterning [16]. Observations in *ktu*-mutant mice revealed that the PCP protein, Vangl1, localized asymmetrically in ependymal and tracheal epithelial cells, while the alignment of basal bodies only differed from wild-type mice in brain ependymal cells, suggesting that ciliary motility was required in the alignment of brain ependymal cells, but not for airway cilia [17].

There are other possible mechanisms, in addition to ciliary motility defects, as causal factors of the phenotypes in SPAG6-mutant mice. In multi-ciliated cells, basal bodies are replicated deep within the cytoplasm, and their apical movement and docking are thought to involve regulated actin assembly [18], [19] and vesicle trafficking [20]. Indeed, actin is enriched at the apical surface of ciliated epithelial cells [21], [22]. Disruption of the actin cytoskeleton blocks basal body migration and ciliogenesis [19]. In this case, the ciliogenesis defect is associated with the failure of basal body docking at the apical plasma membrane. *Spag6*-deficient cells may have a disrupted actin cytoskeleton affecting basal body docking, which traps some basal bodies inside the cytoplasm where they are degraded, with the result that fewer cilia develop in the brain ependymal and tracheal epithelial cells.

A recent study demonstrated that silencing of the *Spag6* gene in Xenopus larvae gives rise to disruption of orientation of basal bodies, suggesting that the planar cell polarity mechanism might be involved [14]. PCP refers to the polarization of a field of cells within the plane of a cell sheet [23]. It is a downstream branch of Wnt signaling [24]. This form of polarization is required for diverse cellular processes in vertebrates, including convergent extension (CE) [25] and the establishment of PCP in epithelial tissues and ciliogenesis [26], [27]. In multi-ciliated cells, planar polarity is present in two distinct models, termed rotational polarity and tissue-level polarity [28], [29]. The former refers to the alignment of the basal bodies within each multi-ciliated cell, and the latter to the coordination of many multi-ciliated cells across the tissue. SEM and high magnification TEM studies clearly demonstrated that *Spag6*-deficient epithelial cells in the brain and trachea lost rotational polarity. Alternatively, SPAG6 may cause ciliogenesis defects through a role in basal bodies. Pearson et al. reported that SPAG6 was present in newly assembled basal bodies [30], and SPAG6 localizes to the center of the transition zone at the site of central pair assembly [31].

Several recent studies revealed that the PCP signaling cascade is a central regulator of the orientation of cilia-mediated fluid flow. Disruption of core PCP genes, including the Dishevelled (Dvl1), Celsr2 and Celsr3 resulted in a randomization of rotational polarity [32], [33], [15]. PCP signaling also controls the tissue-level polarity of multi-ciliated cells, PCP proteins, van Gogh-like 2 (Vangl2) and Frizzled are in this case [29]. A more recent study indicated that PCP proteins, including Vangl1, Vangl2, Prickle2 (PK2), Dishevelled1 (Dvl1) and Dvl2 localize asymmetrically to the tracheal epithelial cell cortex [34].

Little is known about the regularity of orientation of basal bodies in multi-ciliated cells. Previous studies of multi-ciliated cells suggested that microtubules attached to the basal feet link basal bodies to one another, and also to the apical junctions [35]. The classic planar polarity in the Drosophila wing epithelial cells is also associated with sub-apical microtubules [36], [37]. These microtubules are planar polarized, with their plus ends enriched at the distal face of cells, where Dvl1 and Frizzled localize. It appears that the apical microtubule network is an upstream regulator of PCP signaling [38], [39]. It is suggested that a similar planar polarized web of microtubules may also influence planar polarity of basal bodies. In addition, basal bodies in multi-ciliated cells make complex connections to both actin and cytokeratin networks, and these may be involved in polarization [40]. We have previously shown that SPAG6 is a microtubule binding protein [1]. It may play a role in stabilizing the microtubule system. In the absence of SPAG6, sub-apical microtubule stability might be affected, which could result in disruption of polarized PCP distribution, causing the basal bodies to lose their polarized localization.

Defects in mammalian cilia or flagella motility/function caused by mutations in central apparatus genes appear to depend upon the genetic background and cellular context. For instance, mutation of the *Pcdp1* gene results in several phenotypes commonly associated with primary ciliary dyskinesia. Homozygous mutants on a C57BL/6J background develop severe hydrocephalus and mainly die within the first week of life. However, on other genetic backgrounds (129S6/SvEvTac), mice develop either mild or no hydrocephalus with survival to adulthood. The respiratory epithelial cilia have a normal ultrastructure, but beat with reduced frequency. Interestingly, the male mice are infertile, producing sperm with no visible flagella, suggesting that the mechanisms regulating the biogenesis of cilia and flagella are likely to be different [41]. Tracheal epithelial cilia from *Spef2*-defficient mice beat at lower frequency and have a normal 9+2 axonemal structure without apparent defects in the dynein arms, but epididymal sperm lack recognizable axonemal structures [42]. SPAG16L-null mice show no evidence of cilia dysfunction, such as hydrocephalus, sinusitis, and bronchial infection [8], and have tracheal epithelial cells with motile cilia [12]. However, males are infertile due to severe sperm motility defects, even though the sperm have a normal axoneme ultrastructure [8]. In the *Spag17*-mutant mouse, the rapid neonatal demise is associated with a profound respiratory phenotype characterized by immotile cilia and defects in the 9+2 axonemal structure [12]. This is not observed in cilia from knockouts of the *Spag6* and *Spag16* genes. Although the genetic background of mutant mice may significantly influence the phenotypes, the observations summarized above suggest that specific central pair genes may have unique roles in different cell types.

In conclusion, our studies demonstrate that SPAG6 deficiency causes multiple abnormalities in the function of cilia and ciliated cells, including defects in sperm flagellar motility; ciliogenesis; ciliary beat; axoneme orientation; cell morphology and polarity.

## Supporting Information

Figure S1 Analysis of cilia in the trachea epithelial cells and brain ependymal cells by scanning electronic microscopy. Tracheas and brains from wild type and Spag6-deficient mice were processed for SEM. Notice that cilia in the brains (A) and trachea (C) of the wild-type animals sit on the cell surface in a highly ordered state. However, cilia in the ependymal cells (B) and trachea (D) of Spag6-deificent mice appeared to be disordered on the cell surface. Fig. S1 shows cilia in the trachea epithelial cells and brain ependymal cells by scanning electronic microscopy with high magnification.
(TIF)

Figure S2 Circular plots of tracheal epithelial cell basal foot orientation in three individual *Spag6*-deficient (upper) and three individual wild-type mice (lower). Five TEM images were randomly selected from each mouse and basal foot orientations were measured. Arrow direction represents the mean vector of cilium orientation per cell; arrow length is the length of the mean vector, with longer arrows indicating stronger coordination of orientation. *r*_cell_ is the length of mean vector and describes rotational orientation.
(TIF)

Figure S3 Analysis of Vangl2 protein expression level in the lung/trachea by Western blotting. Lungs/tracheas from three wild-type and three *Spag6*-deficient mice were homogenized and Western blotting was performed with anti-Vangl2 antibody, the membrane was striped and re-probed with an anti-actin antibody as a loading control. There was no difference in Vangl2 protein expression level between the wild-type and *Spag6*-deficient mice.
(TIFF)

Figure S4 Examination of Vangl2 and α–tubulin localization in trachea epithelial cells of three-week old mice. The distribution of the PCP protein, Vangl2, and, α-tubulin was examined by immunofluorescence staining. More intense signal was detected in the apical regions in wild-type trachea epithelial cells (arrows in A for Vangl2 and arrowheads in C for α-tubulin). These proteins appeared to be distributed evenly throughout the cytoplasm in cells from *Spag6* mutant mice (dashed arrows in B). In the trachea of wild-type mice, cilia were also intensively stained by an anti-α-tubulin antibody (arrows in C and D).
(TIF)

Figure S5 Examination of α-tubulin localization in trachea epithelial cells in one-week old mice. Distribution of α-tubulin is polarized in the wild-type mice (arrowheads in upper panel). However, the polarized pattern is not seen in the *Spag6*-deficient mice (lower panel), where α-tubulin is evenly distributed throughout the cytoplasm.
(TIF)

Video S1 Trachea ciliary beat observed by video microscopy. Cilia from wild-type mice (*Spag6* knockout littermate) beat at a fast rate, and the beat was coordinated, with all the cilia beating in the same direction at a specific time point.
(AVI)

Video S2 Airway clearance in wild-type mice. Real time video showing the efficiency of ciliated epithelium in moving particles (blood cells) in the trachea. Arrows indicate the direction flow.
(AVI)

Video S3 Cilia from *Spag6*-deficient mice beat at much slower rate, and the beating is largely uncoordinated. At specific time points, some cilia beat in one direction, while others beat in the opposite direction.
(AVI)

Video S4 *Spag6*-deficient mice fail to clear particles from the airway. Uncoordinated cilia from tracheal epithelium failed to generate blood cell flow. Arrows indicate the presence of blood cells stacked at the beginning of tracheal tube. Video is shown in real time.
(AVI)

## References

[1] Sapiro R, Tarantino LM, Velazquez F, Kiriakidou M, Hecht NB, et al (2000) Sperm antigen 6 is the murine homologue of the Chlamydomonas reinhardtii central apparatus protein encoded by the PF16 locus. Biol Reprod 62: 511–518. 10684790 10.1095/biolreprod62.3.511

[2] Smith EF, Lefebvre PA (1996) PF16 encodes a protein with armadillo repeats and localizes to a single microtubule of the central apparatus in Chlamydomonas flagella. J Cell Biol 132: 359–370. 8636214 10.1083/jcb.132.3.359 PMC2120723

[3] Branche C, Kohl L, Toutirais G, Buisson J, Cosson J, et al (2006) Conserved and specific functions of axoneme components in trypanosome motility. J Cell Sci 119 (Pt 16) 3443–3455. 16882690 10.1242/jcs.03078

[4] Straschil U, Talman AM, Ferguson DJ, Bunting KA, Xu Z, et al (2010) The Armadillo repeat protein PF16 is essential for flagellar structure and function in Plasmodium male gametes. PLoS One 5: e12901. 20886115 10.1371/journal.pone.0012901 PMC2944832

[5] House SA, Richter DJ, Pham JK, Dawson SC (2011) Giardia flagellar motility is not directly required to maintain attachment to surfaces. PLoS Pathog 7: e1002167. 21829364 10.1371/journal.ppat.1002167 PMC3150270

[6] Sapiro R, Kostetskii I, Olds-Clarke P, Gerton GL, Radice GL, et al (2002) Male infertility, impaired sperm motility, and hydrocephalus in mice deficient in sperm-associated antigen 6. Mol Cell Biol 22: 6298–6305. 12167721 10.1128/MCB.22.17.6298-6305.2002 PMC134010

[7] Zhang Z, Tang W, Zhou R, Shen X, Wei Z, et al (2007) Accelerated mortality from hydrocephalus and pneumonia in mice with a combined deficiency of SPAG6 and SPAG16L reveals a functional interrelationship between the two central apparatus proteins. Cell Motil Cytoskeleton 64 (5) 360–376. 17323374 10.1002/cm.20189

[8] Zhang Z, Kostetskii I, Tang W, Haig-Ladewig L, Sapiro R, et al (2006) Deficiency of SPAG16L causes male infertility associated with impaired sperm motility. Biol Reprod 74 (4) 751–759. 16382026 10.1095/biolreprod.105.049254

[9] Sisson JH, Stoner JA, Ammons BA, Wyatt TA (2003) All-digital image capture and whole field analysis of ciliary beat frequency. J Microsc 211: 103–111. 12887704 10.1046/j.1365-2818.2003.01209.x

[10] Tsuneoka M, Nishimune Y, Ohta K, Teye K, Tanaka H, et al (2006) Expression of Mina53, a product of a Myc target gene in mouse testis. Int J Androl 29: 323–330. 16533354 10.1111/j.1365-2605.2005.00572.x

[11] Qiu H, Gołas A, Grzmil P, Wojnowski L (2013) Lineage-specific duplications of Muroidea Faim and Spag6 genes and atypical accelerated evolution of the parental Spag6 gene. J Mol Evol 77 (3) 119–29. 24071998 10.1007/s00239-013-9585-9

[12] Teves ME, Zhang Z, Costanzo RM, Henderson SC, Corwin FD, et al (2013) Spag17 is Essential for Motile Cilia Function and Neonatal Survival. Am J Respir Cell Mol Biol 48 (6) 765–772. 23418344 10.1165/rcmb.2012-0362OC PMC3727877

[13] Kunimoto K, Yamazaki Y, Nishida T, Shinohara K, Ishikawa H, et al (2012) Coordinated ciliary beating requires Odf2-mediated polarization of basal bodies via basal feet. Cell 148: 189–200. 22265411 10.1016/j.cell.2011.10.052

[14] Mitchell B, Jacobs R, Li J, Chien S, Kintner C (2007) A positive feedback mechanism governs the polarity and motion of motile cilia. Nature 447: 97–101. 17450123 10.1038/nature05771

[15] Guirao B, Meunier A, Mortaud S, Aguilar A, Corsi JM, et al (2010) Coupling between hydrodynamic forces and planar cell polarity orients mammalian motile cilia. Nat Cell Biol 12: 341–350. 20305650 10.1038/ncb2040

[16] Appelbe OK, Bollman B, Attarwala A, Triebes LA, Muniz-Talavera H, et al (2013) Disruption of the mouse Jhy gene causes abnormal ciliary microtubule patterning and juvenile hydrocephalus. Dev Biol 382 (1) 172–85. 23906841 10.1016/j.ydbio.2013.07.003 PMC3783533

[17] Matsuo M, Shimada A, Koshida S, Saga Y, Takeda H (2013) The establishment of rotational polarity in the airway and ependymal cilia: analysis with a novel cilium motility mutant mouse. Am J Physiol Lung Cell Mol Physiol 304 (11) L736–45. 23525783 10.1152/ajplung.00425.2012

[18] Dawe HR, Farr H, Gull K (2007) Centriole/basal body morphogenesis and migration during ciliogenesis in animal cells. J Cell Sci 120: 7–15. 17182899 10.1242/jcs.03305

[19] Boisvieux-Ulrich E, Lainé MC, Sandoz D (1990) Cytochalasin D inhibits basal body migration and ciliary elongation in quail oviduct epithelium. Cell Tissue Res 259: 443–454. 2317839 10.1007/BF01740770

[20] Sorokin SP (1968) Reconstructions of centriole formation and ciliogenesis in mammalian lungs. J Cell Sci 3: 207–230. 5661997 10.1242/jcs.3.2.207

[21] Park TJ, Haigo SL, Wallingford JB (2006) Ciliogenesis defects in embryos lacking inturned or fuzzy function are associated with failure of planar cell polarity and Hedgehog signaling. Nat Genet 38: 303–311. 16493421 10.1038/ng1753

[22] Pan J, You Y, Huang T, Brody SL (2007) RhoA-mediated apical actin enrichment is required for ciliogenesis and promoted by Foxj1. J Cell Sci 120: 1868–1876. 17488776 10.1242/jcs.005306

[23] Goodrich LV, Strutt D (2011) Principles of planar polarity in animal development. Development 138: 1877–1892. 21521735 10.1242/dev.054080 PMC3082295

[24] Wallingford JB, Mitchell B (2011) Strange as it may seem: the many links between Wnt signaling, planar cell polarity, and cilia. Gene Dev 25: 201–213. 21289065 10.1101/gad.2008011 PMC3034894

[25] Ybot-Gonzalez P, Savery D, Gerrelli D, Signore M, Mitchell CE, et al (2007) Convergent extension, planar-cell-polarity signalling and initiation of mouse neural tube closure. Development 134: 789–799. 17229766 10.1242/dev.000380 PMC1839770

[26] Dworkin S, Jane SM, Darido C (2011) The planar cell polarity pathway in vertebrate epidermal development, homeostasis and repair. Organogenesis 7: 202–208. 22041517 10.4161/org.7.3.18431 PMC3243033

[27] Wallingford JB (2010) Planar cell polarity signaling, cilia and polarized ciliary beating. Curr Opin Cell Biol 22: 597–604. 20817501 10.1016/j.ceb.2010.07.011 PMC2974441

[28] Mirzadeh Z, Han YG, Soriano-Navarro M, García-Verdugo JM, Alvarez-Buylla A (2010) Cilia organize ependymal planar polarity. J Neurosci 30: 2600–2610. 20164345 10.1523/JNEUROSCI.3744-09.2010 PMC2873868

[29] Mitchell B, Stubbs JL, Huisman F, Taborek P, Yu C, et al (2009) The PCP pathway instructs the planar orientation of ciliated cells in the Xenopus larval skin. Curr Biol 19: 924–929. 19427216 10.1016/j.cub.2009.04.018 PMC2720401

[30] Pearson CG, Giddings TH Jr, Winey M (2009) Basal body components exhibit differential protein dynamics during nascent basal body assembly. Mol Biol Cell 20: 904–914. 19056680 10.1091/mbc.E08-08-0835 PMC2633379

[31] Kilburn CL, Pearson CG, Romijn EP, Janet B, Meehl JB, et al (2007) New *Tetrahymena* basal body protein components identify basal body domain structure. J Cell Biol 178: 905–912. 17785518 10.1083/jcb.200703109 PMC2064616

[32] Park TJ, Mitchell BJ, Abitua PB, Kintner C, Wallingford JB (2008) Dishevelled controls apical docking and planar polarization of basal bodies in ciliated epithelial cells. Nat Genet 40: 871–879. 18552847 10.1038/ng.104 PMC2771675

[33] Tissir F, Qu Y, Montcouquiol M, Zhou L, Komatsu K, et al (2010) Lack of cadherins Celsr2 and Celsr3 impairs ependymal ciliogenesis, leading to fatal hydrocephalus. Nat Neurosci 13 (6) 700–707. 20473291 10.1038/nn.2555

[34] Vladar EK, Bayly RD, Sangoram AM, Scott MP, Axelrod JD (2012) Microtubules enable the planar cell polarity of airway cilia. Curr Biol 22 (23) 2203–2212. 23122850 10.1016/j.cub.2012.09.046 PMC3518597

[35] Sandoz D, Chailley B, Boisvieux-Ulrich E, Lemullois M, Laine MC, et al (1988) Organization and functions of cytoskeleton in metazoan ciliated cells. Biol Cell 63: 183–193. 3060202 10.1016/0248-4900(88)90057-3

[36] Eaton S, Wepf R, Simons K (1996) Roles for Rac1 and Cdc42 in planar polarization and hair outgrowth in the wing of Drosophila. J Cell Biol 135: 1277–1289. 8947551 10.1083/jcb.135.5.1277 PMC2121092

[37] Turner CM, Adler PN (1998) Distinct roles for the actin and microtubule cytoskeletons in the morphogenesis of epidermal hairs during wing development in Drosophila. Mech Dev 70: 181–192. 9510034 10.1016/s0925-4773(97)00194-9

[38] Shimada Y, Yonemura S, Ohkura H, Strutt D, Uemura T (2006) Polarizedtransport of Frizzled along the planar microtubule arrays in Drosophila wing epithelium. Dev Cell 10: 209–222. 16459300 10.1016/j.devcel.2005.11.016

[39] Hannus M, Feiguin F, Heisenberg CP, Eaton S (2002) Planar cell polarization requires Widerborst, a B′ regulatory subunit of protein phosphatase 2A. Development 129: 3493–3503. 12091318 10.1242/dev.129.14.3493

[40] Chailley B, Nicolas G, Lainé MC (1989) Organization of actin microfilaments in the apical border of oviduct ciliated cells. Biol Cell 67: 81–90. 2605375 10.1111/j.1768-322x.1989.tb03012.x

[41] Lee L, Campagna DR, Pinkus JL, Mulhern H, Wyatt TA, et al (2008) Primary ciliary dyskinesia in mice lacking the novel ciliary protein Pcdp1. Mol Cell Biol 28 (3) 949–957. 18039845 10.1128/MCB.00354-07 PMC2223405

[42] Sironen A, Kotaja N, Mulhern H, Wyatt TA, Sisson JH, et al (2011) Loss of SPEF2 function in mice results in spermatogenesis defects and primary ciliary dyskinesia. Biol Reprod 85 (4) 690–701. 21715716 10.1095/biolreprod.111.091132 PMC3184289

