# Supplementary figures and images for: Sperm-Associated Antigen 6 (SPAG6) Deficiency and Defects in Ciliogenesis and Cilia Function: Polarity, Density, and Beat
Source: PLoS One. 2014 Oct 21;9(10):e107271. doi: 10.1371/journal.pone.0107271 (PMC4204823; doi:10.1371/journal.pone.0107271)

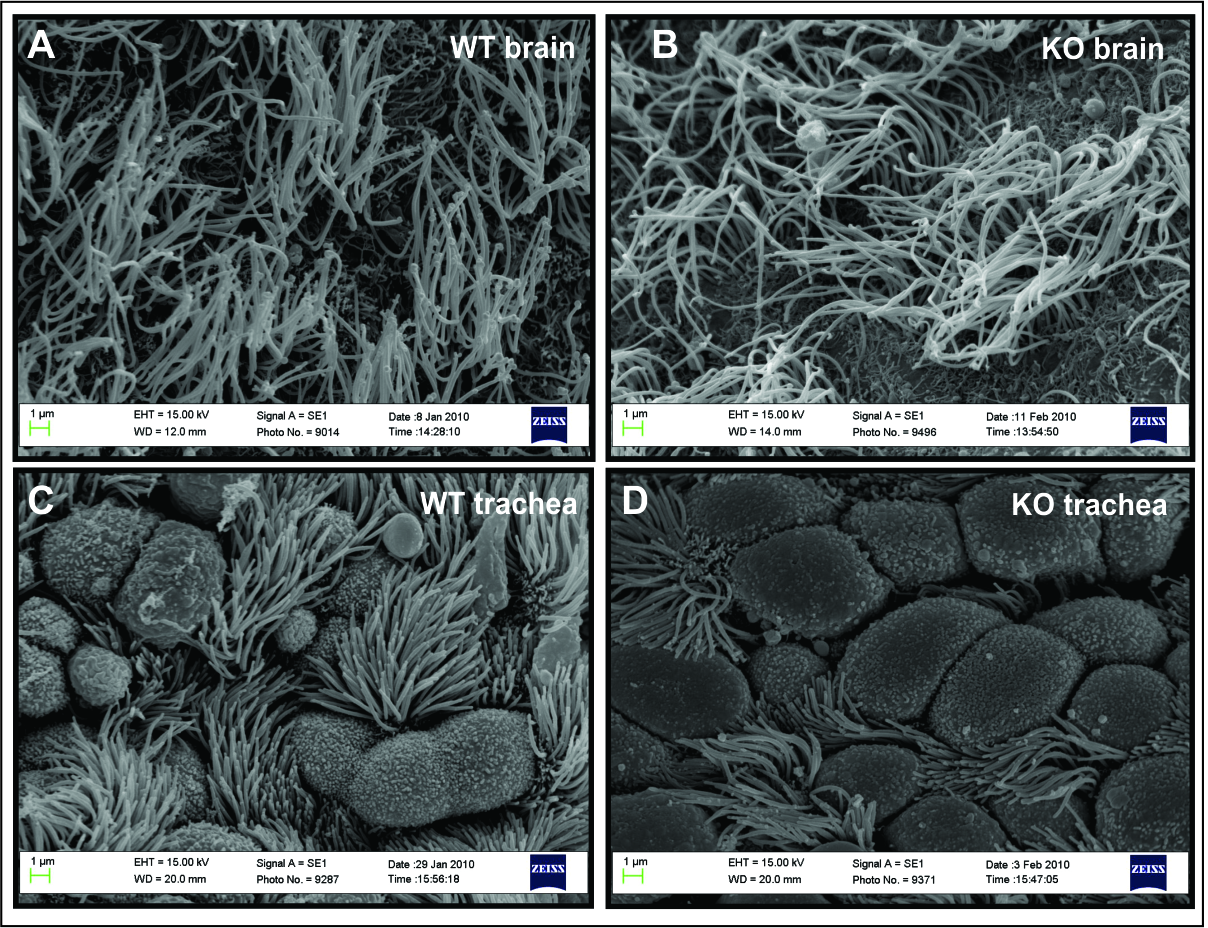

Supplement: Figure S1 — Analysis of cilia in the trachea epithelial cells and brain ependymal cells by scanning electronic microscopy. Tracheas and brains from wild type and Spag6-deficient mice were processed for SEM. Notice that cilia in the brains (A) and trachea (C) of the wild-type animals sit on the cell surface in a highly ordered state. However, cilia in the ependymal cells (B) and trachea (D) of Spag6-deificent mice appeared to be disordered on the cell surface. Fig. S1 shows cilia in the trachea epithelial cells and brain ependymal cells by scanning electronic microscopy with high magnification. (TIF) [file pone.0107271.s001.tif]

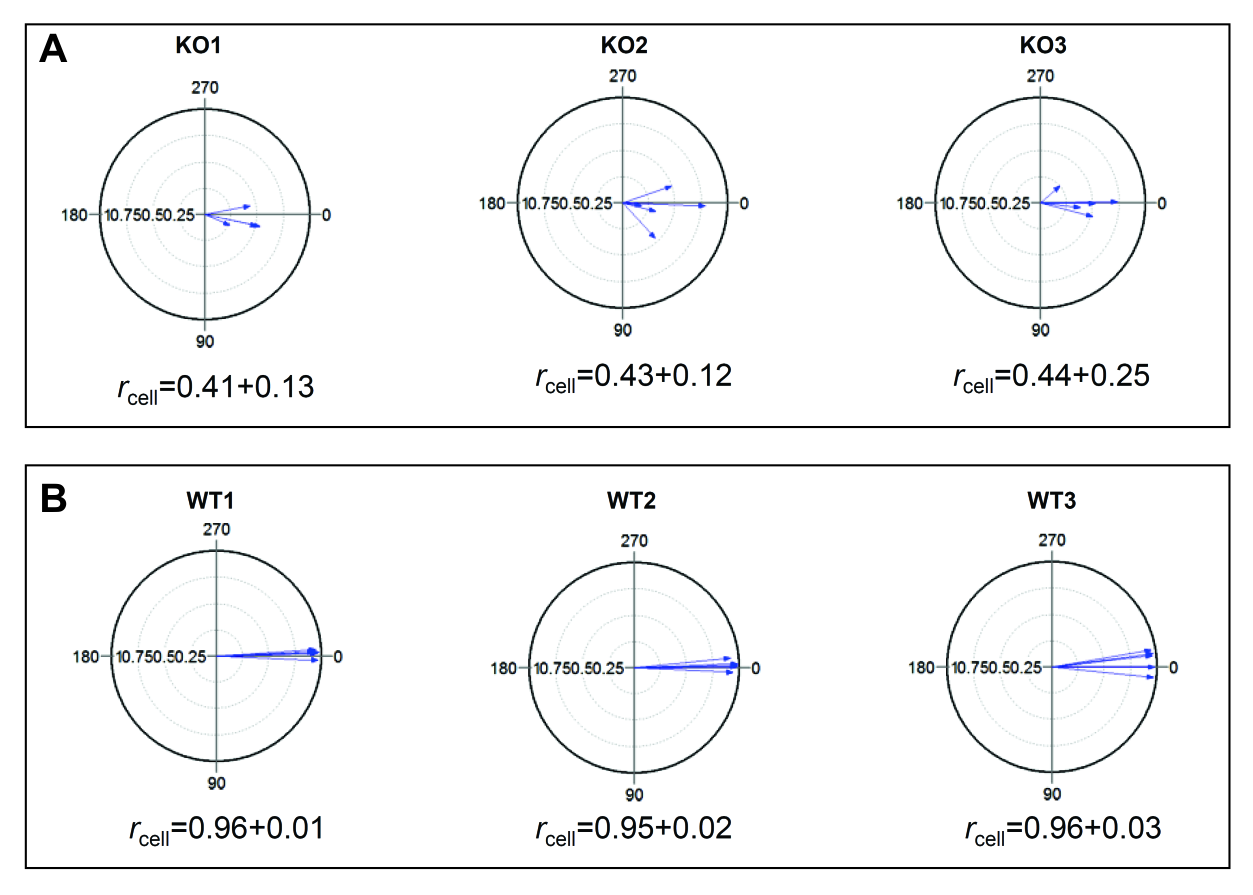

Supplement: Figure S2 — Circular plots of tracheal epithelial cell basal foot orientation in three individual Spag6-deficient (upper) and three individual wild-type mice (lower). Five TEM images were randomly selected from each mouse and basal foot orientations were measured. Arrow direction represents the mean vector of cilium orientation per cell; arrow length is the length of the mean vector, with longer arrows indicating stronger coordination of orientation. r cell is the length of mean vector and describes rotational orientation. (TIF) [file pone.0107271.s002.tif]

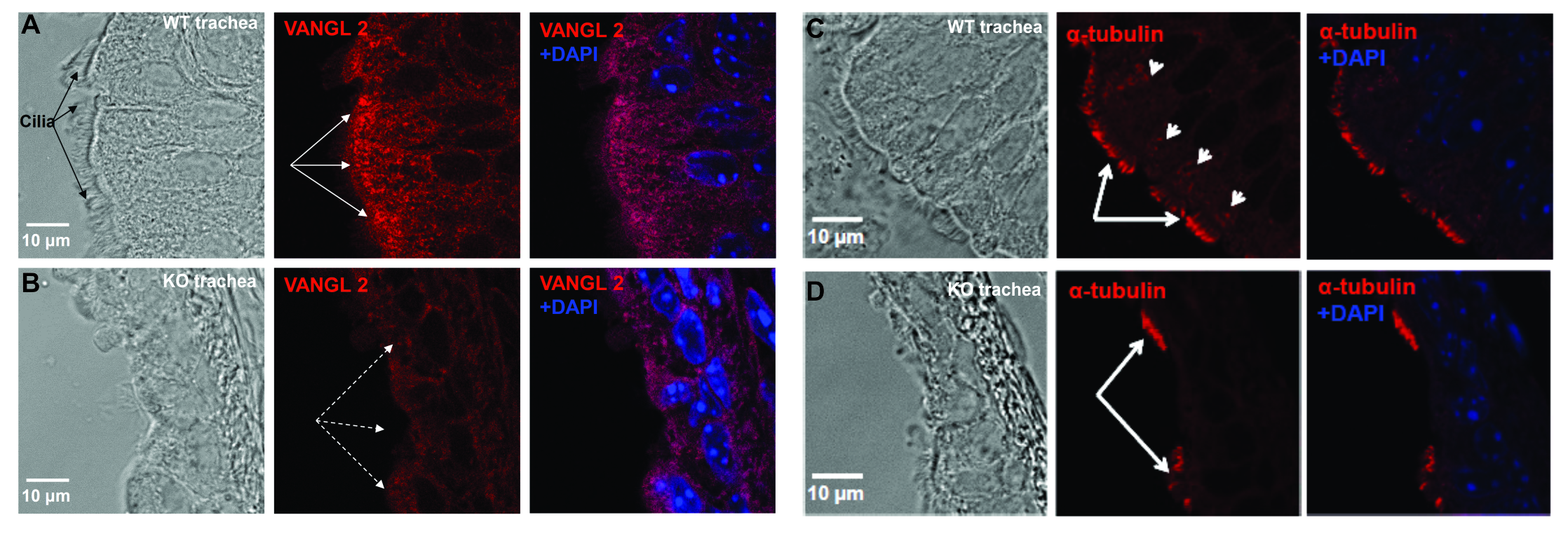

Supplement: Figure S4 — Examination of Vangl2 and α–tubulin localization in trachea epithelial cells of three-week old mice. The distribution of the PCP protein, Vangl2, and, α-tubulin was examined by immunofluorescence staining. More intense signal was detected in the apical regions in wild-type trachea epithelial cells (arrows in A for Vangl2 and arrowheads in C for α-tubulin). These proteins appeared to be distributed evenly throughout the cytoplasm in cells from Spag6 mutant mice (dashed arrows in B). In the trachea of wild-type mice, cilia were also intensively stained by an anti-α-tubulin antibody (arrows in C and D). (TIF) [file pone.0107271.s004.tif]

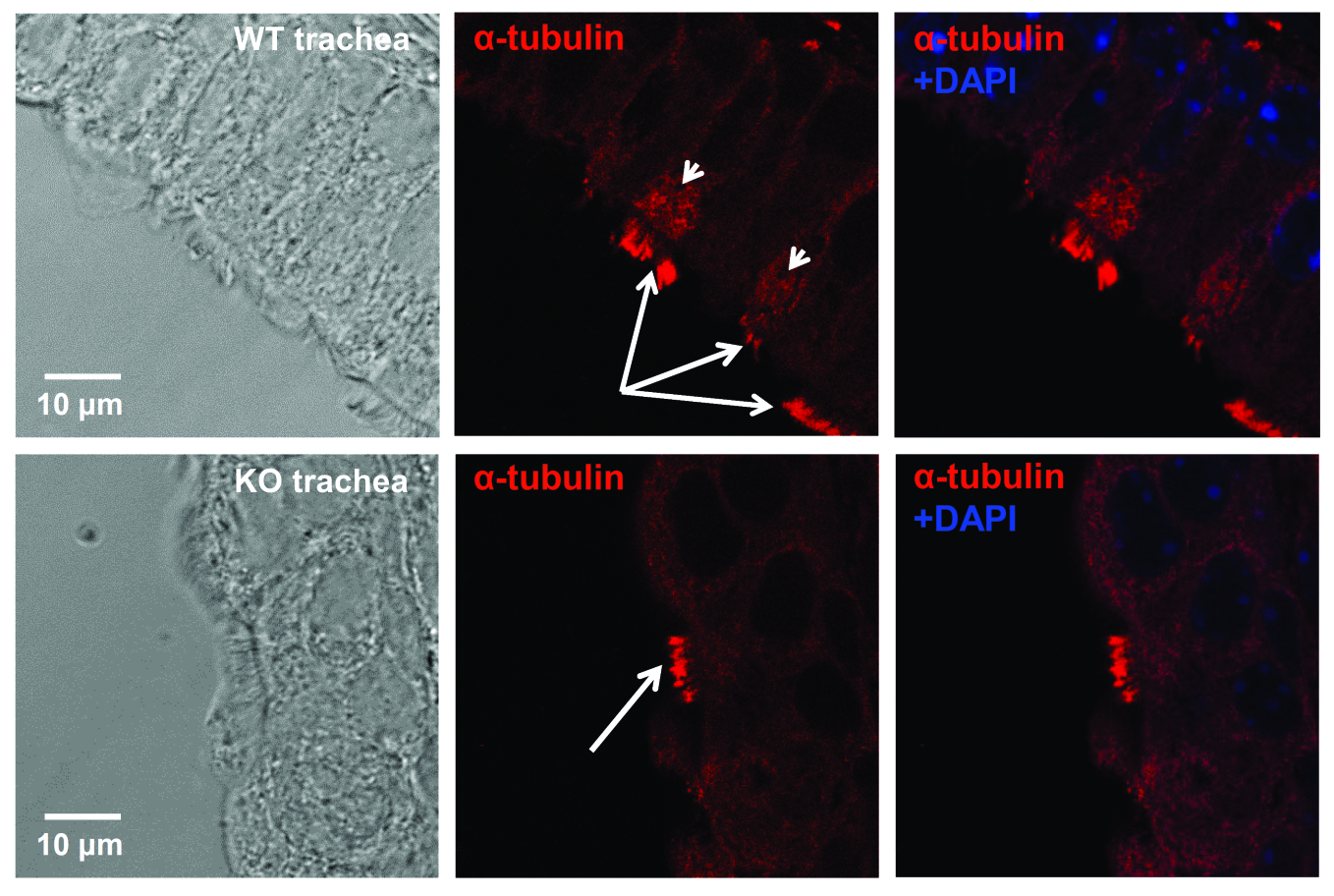

Supplement: Figure S5 — Examination of α-tubulin localization in trachea epithelial cells in one-week old mice. Distribution of α-tubulin is polarized in the wild-type mice (arrowheads in upper panel). However, the polarized pattern is not seen in the Spag6-deficient mice (lower panel), where α-tubulin is evenly distributed throughout the cytoplasm. (TIF) [file pone.0107271.s005.tif]
